# Low Dose Antigen Exposure for a Finite Period in Newborn Rats Prevents Induction of Mucosal Tolerance

**DOI:** 10.1371/journal.pone.0051437

**Published:** 2012-12-12

**Authors:** Rachelle M. Buchanan, Sherry Tetland, Heather L. Wilson

**Affiliations:** Vaccine and Infectious Disease Organization, University of Saskatchewan, Saskatoon, Canada; Charité, Campus Benjamin Franklin, Germany

## Abstract

**Background:**

In adult rats, initial exposure to antigens by a mucosal route triggers tolerance such that any subsequent re-exposure, even by a systemic route, results in suppression of immunity. The newborn’s gut is semi-permeable for a finite period to allow maternal antibodies to enter the newborn’s circulation. We propose that antigens introduced in extreme early life can readily traverse the gut wall and therefore circumvent induction of mucosal tolerance.

**Methodology/Principle Findings:**

Rat pups were gavaged with low-doses of ovalbumin (OVA; oral exposure group) or saline (parenteral control group) every second day for several weeks followed by an intraperitoneal (i.p.) injection at 1 month of age. When gavage was initiated the day after birth, newborn oral exposure pups responded with significantly higher anti-OVA IgA, IgM, IgG2a, and IgG1 titres in their serum and anti-OVA IgA, IgG2a and IgG1 titres in their lungs compared to negative control pups. Oral exposure alone failed to induce immunity. Pups exposed to the same treatment regimen starting at 14 days of age showed induction of mucosal tolerance after i.p. immunization. Newborn oral exposure groups subjected to secondary i.p. immunization responded with significantly increased humoral immunity in lung and sera suggesting that once antigen-specific mucosal tolerance if circumvented, it persists. Lymphocytes derived from mesenteric lymph node cells re-simulated with OVA *ex vivo*, from newborn oral exposure pups exposed to secondary immunization produced significantly higher IFN-γ expression and lymphocyte proliferation relative to control pups indicating prevention of tolerance in the cell-mediated immune system.

**Conclusions/Significance:**

This work demonstrates that newborns may be uniquely qualified to prevent induction of mucosal tolerance to oral antigens. These results should be further explored to establish whether prevention of tolerance by early life oral vaccination can be exploited to prime for mucosal as well as systemic immunity and thus protect this susceptible population against infectious diseases.

## Introduction

Mucosal tolerance is a suppressive mechanism designed to prevent local and peripheral overreaction to innocuous antigens [1], [2]. Far from being a passive or lack of response, mucosal tolerance is a major immunological process taking place continuously at all mucosal sites. Through antigen exclusion, locally produced SIgA or SIgM bind antigens to mask their epitopes, thus preventing an inflammatory response or their binding prevents microbial colonization and penetration of the gut wall [3]. In contrast, mucosal tolerance is a suppressive mechanism designed to avoid local and peripheral overreaction to innocuous antigens [1], [2]. Mucosal DCs sample the luminal environment, traffic to the MLNs, and present the antigen to cognate T cells [4], [5], [6], [7]. DCs play an active role in inducing tolerance through mechanisms which include retinoic acid, vitamin D, IL-10, TGF-β, and indoleamine-2,3,-dioxygenase (reviewed in [8]). In the MLNs, Treg cells undergo differentiation and home back to the inductor site to induce and/or maintain antigen-specific mucosal tolerance [6]. Factors contributing to induction of mucosal tolerance include how antigens are presented to lymphocytes, the host’s immunological maturity at time of exposure, the timing and the frequency of exposure, and the nature of the antigen [9], [10], [11], [12]. A hallmark of oral tolerance is that re-exposure to the antigen, even by systemic routes such as intraperitoneal injection, results in non-responsiveness rather than induction of immunity. Put another way, if a host’s initial exposure to an antigen has been through the oral route and leads to induction of tolerance, it may be difficult to generate an immune response to this antigen in the future.

Despite the overwhelming propensity to respond to an oral antigen with tolerance, oral vaccines are highly sought because of their ease of administration, they are needle-free and therefore present reduced risk of transmitting infections, and there is less need for qualified personnel to administer the vaccine. Moreover, an estimated 90% of all infectious pathogens invade through the mucosal surfaces and therefore mucosal vaccines offer the potential to control pathogens at their point of entry. A significant challenge in induction of oral immunity is that the antigen must be effectively delivered to gut-associated lymphoid tissue (GALT). Several physical barriers prevent antigen/pathogen contact with GALT and penetration of the gut wall such as mucous production, peristaltic movement of the gut, secretion of natural antibacterial substances such as lysozyme and host defense peptides which protect the intestinal surface against bacterial penetration, and the extreme pH environment of the stomach and the protease rich environment of the small intestine which compromise the immunogenicity of ingested antigens [13], [14].

The gut of the newborn is uniquely designed to be ‘semi-permeable’ or leaky for a limited time to allow maternal antibodies to traverse the gut wall in an immunologically-intact form [15], [16], [17]. ‘Gut closure’, the process whereby the gut wall is no longer semi-permeable to macromolecules, occurs within a few days after birth in ruminants [18] [18] and pigs [19], but it does not occur until after weaning (3 weeks) in rats and mice [20], [21], [22]. In humans, a considerable amount of ‘gut-closure’ occurs before birth and within a few days after birth but it may in fact take up to 2 years to reach the same level of impermeability that is observed in the adult gut [23], [24]. We submit that antigens introduced prior to ‘gut-closure’ may be better able to penetrate the gut wall. From here, they can interact with DCs within the sub-epithelial dome which can then present antigens to T cells within the Peyer’s Patch or intestinal lymphoid follicles which can function as sites for induction of mucosal immune responses, rather than being taken up by tolerogenic mucosal DCs which migrate preferentially the MLNs [25], [26].

The purpose of this study was to evaluate whether oral gavage of newborns prevented antigen-specific induction of cell-mediated and antibody-specific mucosal tolerance.

## Materials and Methods

### Ethics Statement

This work was approved by the University of Saskatchewan’s Animal Research Ethics Board (#19940212), and adhered to the Canadian Council on Animal Care guidelines for humane animal use. All procedures were designed to provide the best possible scientific methodologies available with the least discomfort to the animals. All techniques, including the gavage of pups, were refined to provide for maximum comfort/minimal stress to the animals.

### Rat Immunization

**Figure 1:**
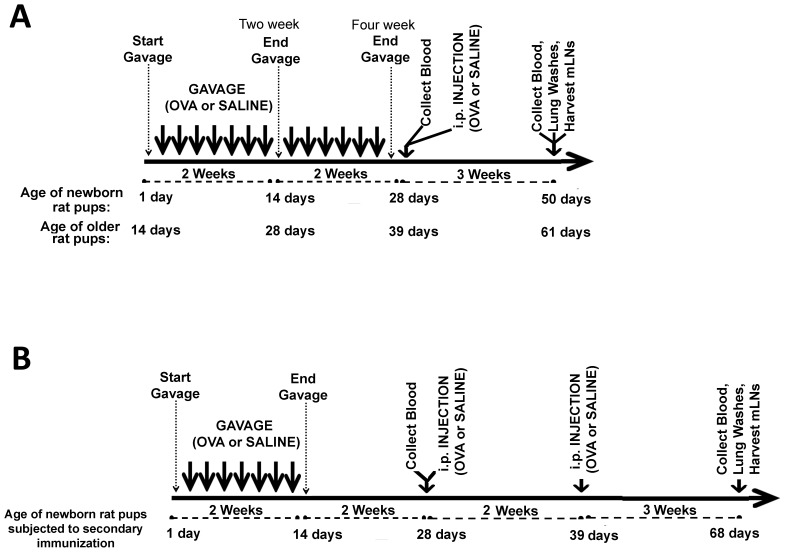
Schematic of experimental conditions for pups. (A) Schematic of experimental conditions for newborn pups and older neonates gavaged and then subjected to one i.p. immunization. Pups were repeatedly gavaged with 1 µg or 0.1 µg OVA or saline starting 1 day after birth (newborns) or starting at 2 weeks of age (older pups). All pups were gavaged for the first 4 days (thinner arrows) followed by gavage every second day (wider arrows) for up to 2 weeks or 4 weeks. Four weeks after initiation of gavage, serum was collected and then pups were i.p. immunized with OVA in IFA or saline. Three weeks later, pups were euthanized and serum, lung lavage and mesenteric lymph nodes were harvested. (B) Schematic of experimental conditions for newborn pups gavaged and then subjected to two i.p. immunizations. Pups were repeatedly gavaged with 1 µg or 0.1 µg OVA or saline starting 1 day after birth for the first 4 days followed by gavage every second day for up to 2 weeks. Four weeks after initiation of gavage, serum was collected and then pups were i.p. immunized with OVA in IFA or saline. Two weeks later, serum was collected and then pups were i.p. immunized a second time with OVA in IFA or saline.

Female Wistar rats (Charles River Laboratories, Inc., Montreal, PQ, Canada) purchased at 14 days gestation were housed in separate cages at VIDO-Intervac for one week prior to whelping and fed standard diet with *ad libitum* access to water and chow. The immunization strategy per group is detailed in Table 1. Briefly, each litter was divided into four experimental groups with no cross-fostering of pups between litters. A gauge×25 mm sterile feeding tube (gavage needle; Instech Solomon, Plymouth Meeting, PA) was gently inserted into the throat and a 25 µl volume containing 1 or 0.1 µg OVA (Sigma-Aldrich Canada Ltd, Oakville, ON, Canada) was administered. (Endotoxin levels in OVA was determined to be 8,000 U/ml using the Limulus Amebocyte Lysate enzymatic assay QCL-1000 (Lonza Group Ltd, Basel, Switzerland) according to the manufacturer’s instructions). This immunisation was repeated daily for four days and then every second day until day 14 or day 28 as indicated. Pups were not gavaged daily to prevent extensive irritation to the throat. For newborn pups, gavage was initiated the day after birth. For the older neonatal groups, gavage was initiated 14 days after birth. After 21 days, all pups were weaned from their mothers. Pups were subjected to i.p. immunization with saline or OVA (200 µg/100 µl total volume; Sigma-Aldrich Canada Ltd.) with Incomplete Freund’s Adjuvant (IFA; Sigma-Aldrich Canada Ltd.) as indicated (Fig. 1A, 1B). Pups subjected to secondary i.p. immunization were re-immunized with the same dose of OVA with IFA 2 weeks after the primary immunization. To generate groups of pups which responded with a primary immune response to OVA, pups were gavaged with saline then injected with OVA via i.p. (i.e. the parenteral control groups; Group P). For all negative control groups, pups were orally gavaged and i.p.-injected with saline. Rats were euthanized 3 weeks post i.p. immunization with an over-dose of isofluorane (AErrane, Baxter Corporation, Mississauga, ON, Canada) followed by cervical dislocation. At time of death, sera, lung washes, and mesenteric lymph nodes were harvested. Experiments were repeated twice. Pups <1 week old were designated as newborns and older neonates were considered >13 days of age [27].

**Table 1:** Details of immunization regimen.

Newborn Groups	Gavage	Duration of Gavage	i.p. immunization	Number of pups	Figures
Saline control group (C)	Saline	2 weeks	Saline	n = 5	2 A–C, 3 A–D, 5 A, 6 A
Parenteral control group (P)	Saline	2 weeks	200 µg OVA and IFA	n = 6	
Oral exposure group (D1)	1 µg OVA	2 weeks	200 µg OVA and IFA	n = 7	
Oral exposure group (D2)	0.1 µg OVA	2 weeks	200 µg OVA and IFA	n = 6	
Saline control group (C)	Saline	4 weeks	Saline	n = 5	
Parenteral control group (P)	Saline	4 weeks	200 µg OVA and IFA	n = 6	
Oral exposure group (D1)	1 µg OVA	4 weeks	200 µg OVA and IFA	n = 7	
Oral exposure group (D2)	0.1 µg OVA	4 weeks	200 µg OVA and IFA	n = 6	
Older Neonate Groups	Gavage	Duration of Gavage	i.p. immunization	Number of pups	Figures
Saline control group (C)	Saline	2 weeks	Saline	n = 5	2 D–F, 3 E–H, 5 B, 6 B
Parenteral control group (P)	Saline	2 weeks	200 µg OVA and IFA	n = 6	
Oral exposure group (D1)	1 µg OVA	2 weeks	200 µg OVA and IFA	n = 7	
Oral exposure group (D2)	0.1 µg OVA	2 weeks	200 µg OVA and IFA	n = 6	
Secondary i.p. immunization Group	Gavage	Duration of Gavage	Primary and secondary i.p. immunization	Number of pups	Figures
Saline control group (C)	Saline	2 weeks	Saline	n = 6	4 A–F, 5 C, 6 C
Parenteral control group (P)	Saline	2 weeks	200 µg OVA and IFA	n = 8	
Oral exposure group (D1)	1 µg OVA	2 weeks	200 µg OVA and IFA	n = 7	
Oral exposure group (D2)	0.1 µg OVA	2 weeks	200 µg OVA and IFA	n = 7	

### Sample Collection

Blood samples were collected from dams before vaccination. In order to evaluate immunity in the offspring following vaccination, serum samples of neonatal pups were collected 2, 4, 6 and 8 weeks post vaccination. Only the data collected from the pups prior to i.p. immunization and at day of animal harvest are reported. All blood samples were centrifuged (4547×g) and serum was stored at −20°C. Bronchoalveolar lavage (BAL) fluid was obtained on day of harvest as detailed in [28].

### Enzyme-linked Immunosorbent Assays (ELISA)

To measure OVA-specific IgG1 and IgG2a titres in blood serum and lung lavages, ELISAs were performed as previously described [29]. To measure OVA-specific IgA and IgM, ELISAs were performed as indicated with the exception that after diluted rat serum or lung lavage samples were added to the wells at 100 µl/well and incubated overnight at 4°C, wells were washed again with Tris-buffered saline (TBS) with 1% Tween-20 (TBST, Sigma-Aldrich Canada Ltd.) and horseradish peroxidase (HRPO)-conjugated goat-anti rat IgA (Bethyl Laboratories, 1/10,000, A100-102P) or HRPO-conjugated goat-anti rat IgM (Bethyl Laboratories, 1/5,000, A100-100P2) was added to separate wells in a 100 µl volume and incubated for 1 h at room temperature (RT). Wells were washed 5 times in TBST then 3,3′,5,5′-Tetramethylbenzidine (Sigma-Aldrich Canada Ltd.) was incubated for 20 min at room temperature followed by the addition of 50 µl 2 N sulphuric acid to arrest the reaction. Assays were performed in duplicate with mean values being reported for each biological replicate. Titres were reported as the reciprocal of the highest dilution that gave a positive OD reading.

### Lymph Node Cytokine ELISAs and Lymphocyte Proliferative Responses

To measure cell-mediated immune responses, draining mesenteric lymph nodes were isolated, fat was dissected from the lymph node before placing the tissue in phosphate-buffered saline containing 0.1% EDTA (PBSA). The lymph node was minced with a scalpel blade and the resulting cell suspension was filtered through a 40-µm cell strainer (BD Falcon, Mississauga, ON, Canada), washed in PBSA and resuspended in culture medium at a final concentration of 6×10^5^ viable cells/ml [30]. Cells were stimulated with 10 µg/mL OVA or media for 18–20 hr. For IFN-γ and IL-4 cytokine ELISAs, cells were isolated as above then stimulated with 10 µg/mL OVA or media. Culture supernatants were evaluated for cytokines after 96 hr stimulation as previously described [31].

### Statistical Analysis

All statistical analyses and graphing were performed using GraphPad Prism 5 software (GraphPad Software, San Diego, CA). Statistical analysis was performed as described previously [32]. Briefly, as outcome variables were found to be not distributed normally, differences within groups were examined using Kruskal-Wallis test or Mann–Whitney tests as appropriate. Differences were considered significant if p<0.05.

## Results and Discussion

### Experimental Design

Experiments were designed to investigate the immunological consequences of oral gavage with OVA when animals were administered the antigen in the immediate or later perinatal period and re-challenged with OVA via i.p. immunization 4 weeks later. Previously, we performed a time and dose course analysis where-in newborns were gavaged with from 0.1 µg to 10 mg OVA for either 4 days, 7 days, or 14 days beginning the day after birth. Serosal and mucosal anti-OVA antibodies were consistently highest in the newborn group exposed for 2 weeks with the lowest dose and therefore these parameters were chosen for further study (data not shown). Figures 1A and 1B detail the experimental design of the trials and the age of the pups for each immunization and at time of harvest.

Table 1 summarizes the experimental treatments, ages of the pups at time of gavage, and corresponding figures. Newborn oral exposure pups were gavaged beginning 1 day after for 2 weeks or 4 weeks and i.p. immunized with OVA 2 weeks later birth (depicted in Fig. 1A). Pups were euthanized 3 weeks later. Pups which comprised the negative control group were gavaged and i.p. immunized with saline. Newborn pups gavaged for 2 weeks then i.p. immunized twice are identified as the secondary immunization group (depicted in Fig. 1B). Older neonatal groups were gavaged for 2 weeks starting at 14 days of age, i.p. immunized 2 weeks later at age 39 days and then euthanized on day 61. Pups which comprised the parenteral control groups were gavaged with saline starting the day after birth (newborn) or at day 14 (older neonates) for 2 weeks, respectively, followed by an injection via the i.p. route with OVA 2 weeks later. Parenteral control pups were reported to have produced an immune response if the group’s anti-OVA antibody titres were significantly higher than the anti-OVA antibody titres reported for the negative control group. Mucosal antibody-dependent immune responses were measured in BAL fluid as a representative mucosal compartment. Anti-OVA antibody titres for the oral exposure group that were significantly lower than the titres reported for the age-matched parenteral control groups were reported as mucosal tolerance. Anti-OVA antibody titres for the oral exposure groups which were significantly higher than or statistically similar to the titres reported for the age-matched parenteral control group, we reported that mucosal tolerance was prevented. To establish whether oral immunization influenced cell-mediated immunity, lymphocyte proliferation and IFNγ and IL-4 cytokine expression were monitored in lymphocytes harvested from the draining mesenteric lymph nodes.

### Newborns Respond to Oral Gavage with OVA-specific Antibodies in the Mucosa

**Figure 2:**
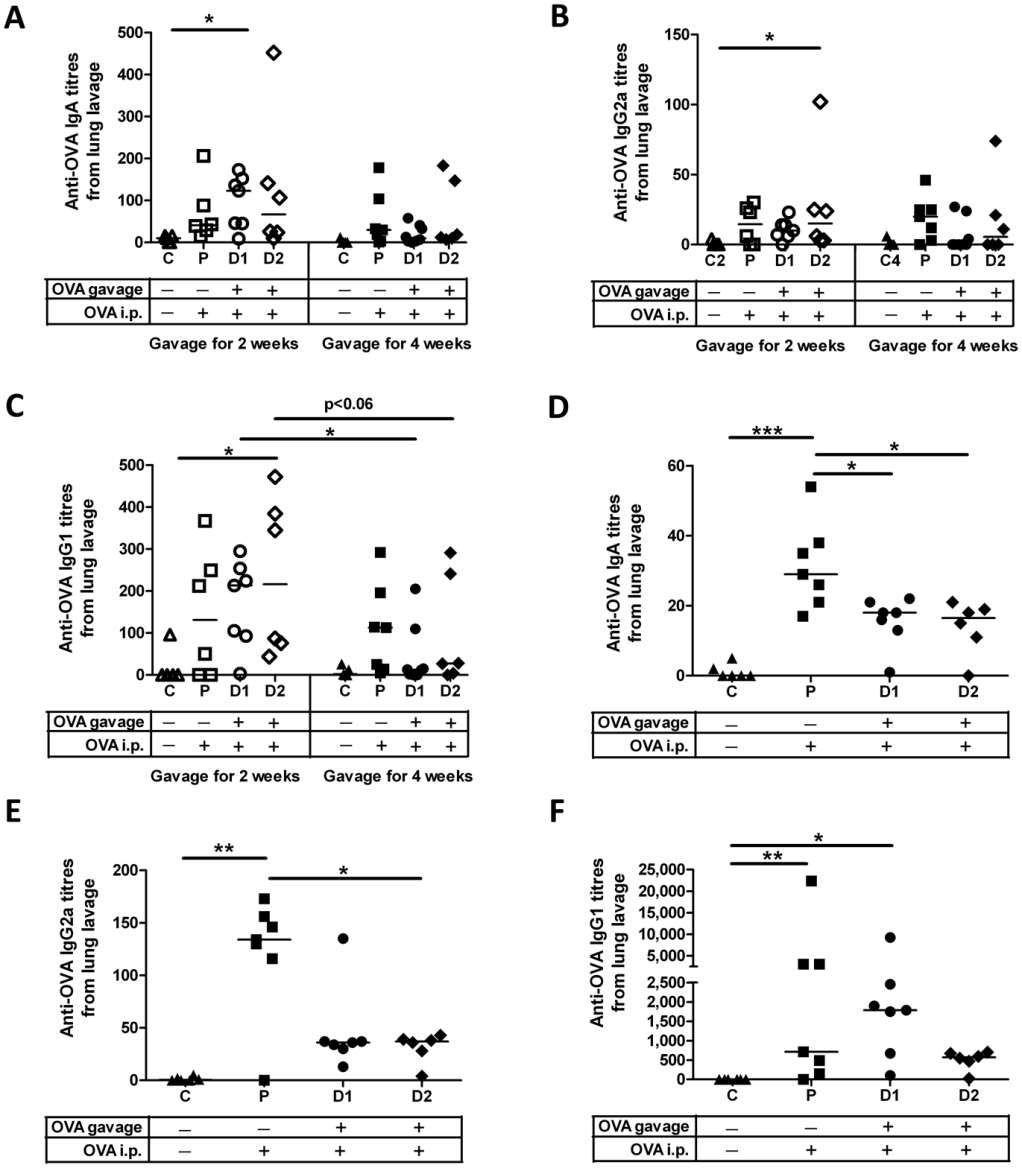
Oral antigen exposure in newborns triggers antibody production at distal mucosal sites. Pups were treated as detailed in Fig. 1A. Bronchoalveolar (lung) lavages were collected at the time of death. Anti-OVA IgA titres (A), IgG2a titres (B) and IgG1 titres (C) were assessed for newborns gavaged for 2 weeks or 4 weeks. Anti-OVA IgA titres (D), IgG2a titres (E) and IgG1 titres (F) were assessed for older neonates gavaged for 2 weeks. Data shown are presented as the mean of duplicate titres for individual biological replicates and the horizontal line represents the median value for the group. * p<0.05; ** p<0.01; *** p<0.001.

Due to dissemination of antigen-sensitized precursor B and T lymphocytes from mesenteric lymph nodes, antigen-specific antibodies and cellular responses generated at one mucosal site such as the gut can be detected at anatomically remote and functionally distinct compartments such as the respiratory mucosa [33], [34], [35], [36]. Therefore, to establish whether oral gavage of newborn pups promoted mucosal immunity, we measured anti-OVA antibody titres in lung washes 3 weeks after i.p. immunization. Pups gavaged with 1 µg OVA for 2 weeks (Group D1) responded with significant anti-OVA IgA titres (Fig. 2A, p<0.05) and pups gavaged with 0.1 µg OVA for 2 weeks (Group D2) responded with significant anti-OVA IgG2a (Fig. 2B, p<0.05) and IgG1 titres (Fig. 2C, p<0.05) relative to the newborn negative control pups. When the gavage persisted for 4 weeks, none of the newborn pups responded with significant antibody production in the lung. In fact, pups gavaged with 1 µg OVA (Group D1) for 2 weeks produced significantly higher anti-OVA IgG1 (Fig. 2C, p<0.05) compared to pups gavaged with the same dose for 4 weeks. Pups gavaged with the lower dose for 2 weeks also showed this trend relative to the pups gavaged for 4 weeks (Fig. 2C, Group D2, p<0.06). These kinetic data suggest that the time in which mucosal tolerance can be subverted is limited to less than 1 month after birth in rats and the response is influenced by dose. Anti-OVA IgM was not detected in BAL fluid for any groups under investigation (data not shown). The newborn parenteral control group (Group P) showed a trend towards induction of immunity for all anti-OVA isotypes relative to the control group but these data were not statistically significant. These data indicate that by subjecting newborn pups to persistent oral gavage for 2 weeks, antigen-specific mucosal antibody production was induced suggesting prevention of mucosal tolerance in the lung.

To determine whether it was critical to introduce the gavage regimen in the immediate perinatal period, we repeated the experiment but delayed starting the gavage until the pups were 14 days old. Older neonatal oral exposure pups gavaged with 1 µg OVA (Group D1) produced significant anti-OVA IgG1 (Fig. 2F, p<0.05) titres in BAL fluid relative to age-matched negative control pups but they responded with significantly lower anti-OVA IgA (Fig. 2D, Group D1 p<0.05; Group D2 p<0.05) and IgG2a (Fig. 2E, Group D2 p<0.05) titres compared to parenteral control pups suggesting that if gavage was delayed for just 14 days after birth, mucosal tolerance, not immunity, was induced. In contrast to the newborn parenteral control group, the parenteral older neonate control group responded to OVA i.p. immunization with significant anti-OVA IgA (Fig. 2D, p<0.001), IgG2a (Fig. 2E, p<0.01) and IgG1 (Fig. 2F, p<0.01) titres relative to age-matched control pups. These data suggest that despite being separated in age by only 2 weeks, the neonatal parenteral control group i.p. immunized with OVA at 6 weeks of age were sufficiently mature to mount a robust mucosal immune response, unlike the newborn parenteral control groups which were i.p. immunized at 4 weeks of age.

### Newborns Respond to Oral Gavage by Preventing OVA-specific Systemic Humoral Tolerance

Our next steps involved establishing whether oral antigen exposure in newborns prevented induction of systemic humoral tolerance. Because antibodies from the dams will be present in the pup’s sera in the perinatal period, we monitored the serum anti-OVA IgG1, IgG2a, IgA and IgM titres in the dams 1 week prior to birthing and on the day of weaning. Anti-OVA antibody titres for all serotypes were negligible (data not shown). To further confirm that the pups were not receiving anti-OVA antibodies via passive immunity, serum antibody titres in pups were evaluated for all groups at 1 week of age and all pups showed negligible titres (data not shown).

**Figure 3:**
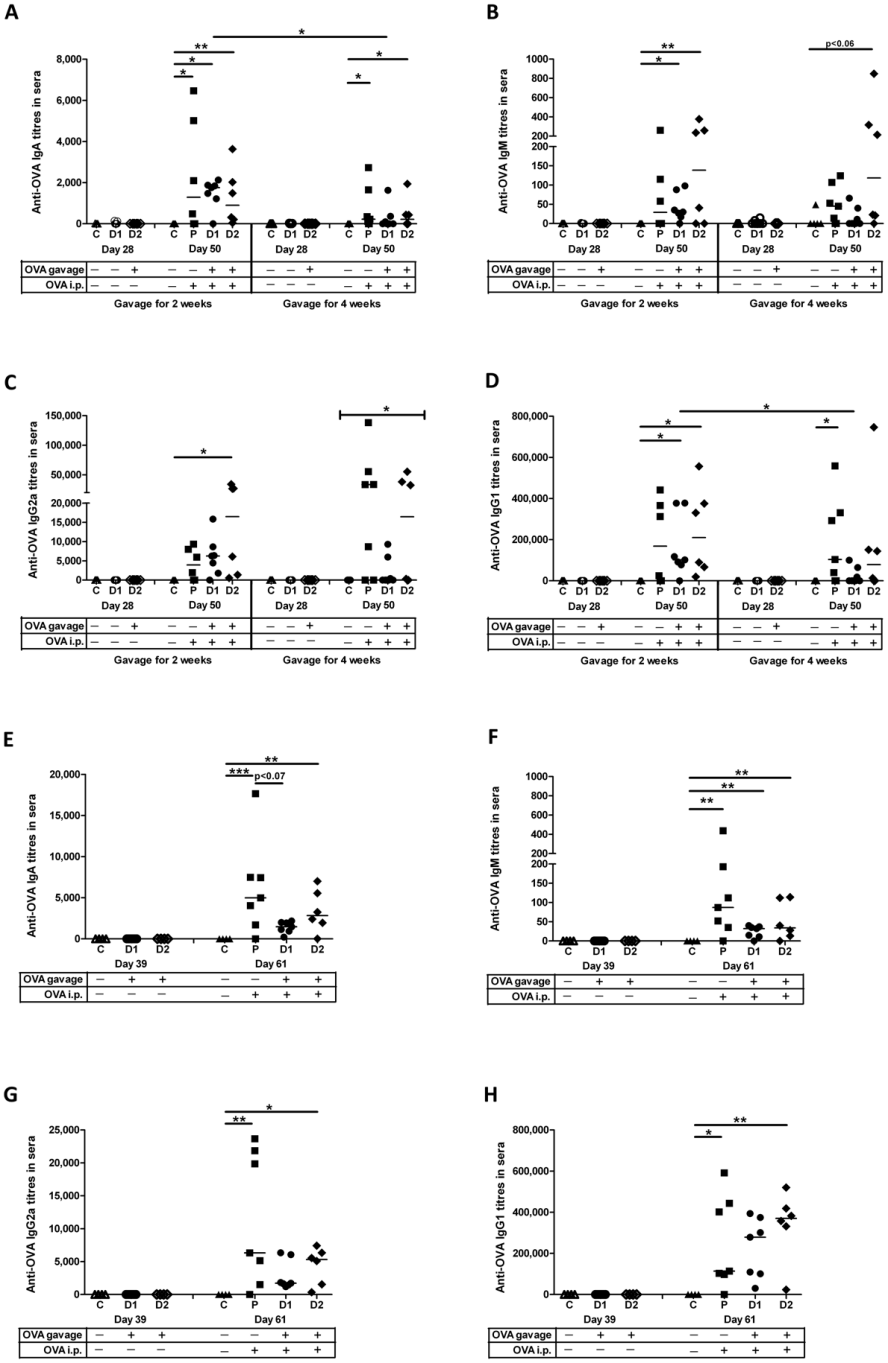
Oral antigen exposure in newborns prevents humoral tolerance in sera. Pups were treated as detailed in Fig. 1A. Anti-OVA IgA titres (A), IgM titres (B), IgG2a titres (C) and IgG1 titres (D) were assessed on day 28 and day 50 for newborns that were gavaged for 2 weeks or 4 weeks. Anti-OVA IgA titres (E), IgM titres (F), IgG2a titres (G) and IgG1 titres (H) were assessed on day 39 and day 61 for older neonates that were gavaged for 2 weeks. Data shown are presented as the mean of duplicate titres for individual biological replicates and the horizontal line represents the median value for the group. * p<0.05; ** p<0.01; *** p<0.001.

Next, we assessed whether newborn pups from the oral exposure groups showed induction of antibodies against OVA in the sera representing subversion of mucosal tolerance. From serum obtain immediately prior to i.p. immunization (day 28), newborn pups gavaged with either 1 or 0.1 µg OVA for 2 or 4 weeks failed to produce significant anti-OVA IgA (Fig. 3A), IgM (Fig. 3B), IgG2a (Fig. 3C) or IgG1 (Fig. 3D) titres indicating that oral exposure itself does not induce immunity. However, when serum from these same pups was evaluated 3 weeks post i.p. immunization (day 50), the group that was gavaged for 2 weeks with 0.1 µg OVA (Group D2) generated significant anti-OVA IgA (Fig. 3A, p<0.01), IgM (Fig. 3B, p<0.01), IgG2a (Fig. 3C, p<0.05) and IgG1 (Fig. 3D, p<0.05) titres relative to the newborn control pups. The group gavaged for 2 weeks with 1 µg OVA (Group D1) also induced significant anti-OVA IgA (Fig. 3A, p<0.05), IgM (Fig. 3B, p<0.05), and IgG1 titres (Fig. 3D, (p<0.05) indicating that persistent oral exposure of OVA to newborns prevented induction of mucosal tolerance. As was observed in the BAL fluid, no significant induction of anti-OVA antibodies relative to control pups of anti-OVA IgM, IgG2a and IgG1 were produced in newborn pups gavaged with either 1 or 0.1 µg OVA for 4 weeks. In fact, pups gavaged for 4 weeks with 1 µg OVA showed significantly lower anti-OVA IgG1 titres (Fig. 3D, Group D1 p<0.05) relative to the pups gavaged with this dose for only 2 weeks. However, the group of newborn pups gavaged for 4 weeks with 0.1 µg OVA (Group D2) did show significant induction of anti-IgA (Fig. 3A, p<0.05) and a trend toward induction of anti-OVA IgM (Fig. 3B, p<0.06) relative to control pups.

**Figure 4:**
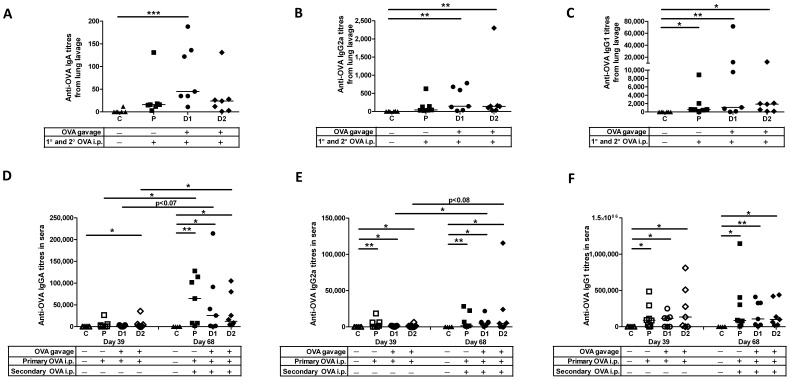
Oral antigen exposure in newborns prevents humoral tolerance in lungs and sera after repeated systemic antigen exposure. Pups were treated as detailed in Fig. 1B. Rats were euthanized and serum, lung lavage and mesenteric lymph nodes were harvested on day 68 after birth. Anti-OVA IgA titres (A,D), IgG2a titres (B, E) and IgG1 titres (C, F) were measured in BAL fluid and sera, respectively. Data shown are presented as the mean of duplicate titres for individual biological replicates and the horizontal line represents the median value for the group. * p<0.05; ** p<0.01; *** p<0.001.

The parenteral control group induced significant anti-OVA IgA (Fig. 3A (2 and 4 weeks), p<0.05), and IgG1 (Fig. 3D (4 weeks), p<0.05) titres relative to the time-matched negative control group. However, it is worth noting that unlike the oral control groups, the parenteral control groups failed to produce significant anti-OVA IgM (Fig. 3B, 2 and 4weeks), anti-OVA IgG2a (Fig. 3C, 2 and 4weeks), anti-OVA IgG1 (Fig. 3D, 2 weeks) relative to control groups. These data suggest that anti-OVA titres produced in the oral exposure groups were not simply due to i.p. immunization and that response to oral antigen priming may contribute to antibody production. Thus, as with the mucosal compartment, the group of newborn pups that were the most successful in subverting mucosal humoral tolerance were the pups gavaged with 0.1 µg OVA for 2 weeks. This time course was continued for the remaining experiments.

When we assessed the serum from older neonatal pups which were gavaged starting at 14 days of age, we observed that the group gavaged for 2 weeks with 1 or 0.1 µg OVA alone prior to i.p. immunization (day 39) failed to induce significant anti-OVA antibodies in serum (Fig. 3E–H). However, the older neonatal oral exposure group gavaged for 2 weeks with 1 or 0.1 µg OVA followed by i.p. immunization (day 61) generated significant anti-OVA IgA (Fig. 3E, Group D2, p<0.01), IgM (Fig. 3F, Group D1 p<0.01, Group D2 p<0.01), IgG2a (Fig. 3G, Group D2 p<0.05), and IgG1 (Fig. 3H, Group D2 p<0.01) titres in sera relative to the age-matched control pups. It is interesting to note that unlike mucosal tolerance which was observed in the lung (Fig. 2 D–E), significant OVA-specific IgA and IgG2a titres were detected in the serum from the pups gavaged starting at 14 days of age (Fig. 3E,G). This limited humoral response observed in the older pup oral exposure groups may be due to the fact that in rats, gut-closure is not complete until after weaning and intestinal permeability significantly tapers off after the second week of life [22]. Therefore, pups gavaged starting on day 14 may have experienced limited transmigration of OVA from the lumen into the gut which induced limited humoral immunity in sera. Importantly, the older neonatal oral exposure group gavaged with 1 µg OVA (Group D1) showed a trend towards reduced anti-OVA IgA (Fig. 3E, p<0.07) and IgM (Fig. 3F, p<0.07) titres in serum relative to the age-matched parenteral control pups indicating induction of mucosal tolerance which is in agreement with the response in the lung. Finally, the older neonatal parenteral control pups generated significant anti-OVA IgA (Fig. 3E, p<0.001), IgM (Fig. 3F, p<0.01), IgG2a (Fig. 3G, p<0.01) and IgG1 (Fig. 3H, p<0.05) titres in serum relative to the older neonatal negative control pups suggesting that despite the fact that they are only 2 weeks older than the newborn group, the older neonatal pups have an immune system that is sufficiently mature to generate a robust systemic immune response to OVA.

### Mucosal Tolerance to OVA Persists into Adulthood

To establish whether mucosal tolerance is maintained over time, newborn pups were gavaged for 2 weeks and i.p. vaccinated at 4 weeks of age as before, and then were i.p. immunized again at 6 weeks of age (Fig. 1B). In lung lavages, newborn pups gavaged with 1 µg and 0.1 µg OVA showed significantly higher anti-OVA IgA (Fig. 4A, D1 p<0.001), IgG2a (Fig. 4B, Group D1 p<0.01; Group D2 p<0.01)) and IgG1 (Fig. 4C, Group D1 p<0.01; Group D2 p<0.05) titres relative to age-matched control pups indicating prevention of mucosal tolerance. These titres were extremely robust when compared to the newborn oral exposure groups in Fig. 3A, 3C and 3D that were subjected to 2 weeks gavage followed by a single i.p. immunization (day 50). Newborn pups vaccinated only via systemic routes (Group P) failed to induce significant titres of anti-OVA IgA (Fig. 4A) or IgG2a (Fig. 4B) in lung lavages but they did produce significant anti-OVA IgG1 titres (Fig. 4C, p<0.05) relative to age-matched controls. None of the groups showed induction of anti-OVA IgM in BAL fluid (data not shown).

Next, we monitored the humoral response in serum over time. When we assessed the serum antibody titres after the primary immunization (day 39), the newborn group of pups gavaged with 0.1 µg OVA (Group D2) showed significant anti-OVA IgA (Fig. 4D, p<0.05), IgG2a (Fig. 4E, p<0.05) and IgG1 (Fig. 4F, p<0.05) titres in sera relative to the newborn control group. These data show excellent agreement with the newborn oral exposure groups in Fig. 3A–D which were also subjected to 2 weeks gavage followed by a single i.p. immunization (day 50). However, unlike in Fig. 3, the group of pups gavaged with the higher dose of OVA (Group D1) or parenteral control group subjected to primary i.p. immunization (day 39) also showed significant induction of anti-OVA IgG2a (Fig. 4E, Group D1 p<0.05; Group P p<0.01) and IgG1 (Fig. 4F, Group P p<0.05) titres relative to their control pups. These discrepancies may be due to an increase of 1 pup per group in Figure 4 compared to Figure 3 which bore out more statistically robust data in the latter figure (See Table 1).

When serum antibody titres were assessed after the secondary immunization (Day 68), all groups showed significant induction of anti-OVA IgA (Fig. 4D, Group P p<0.01; Group D1 p<0.05; Group D2 p<0.05), IgG2a (Fig. 4E, Group P p<0.01; Group D1 p<0.05; Group D2 p<0.05), and IgG1 (Fig. 4F, Group P p<0.05; Group D1 p<0.01; Group D2 p<0.05) titres relative to age-matched controls, respectively. In fact, relative to what was observed in serum harvested after the primary immunization, pups subjected to secondary i.p. immunization showed significantly higher anti-OVA IgA titres in the serum when the newborn pups were gavaged with 0.1 µg OVA (Fig. 4D, Group D2 p<0.05) and when the pups were exclusively immunized by the parenteral route (Group P p<0.05). Likewise, secondary i.p. immunization significantly induced anti-OVA IgG2a titres in serum from the D1 oral exposure groups (Fig. 4E, p<0.05) relative to the titres generated after a single i.p. immunization and these trends were observed in the oral exposure groups for anti-OVA IgA (Fig. 4D Group D1, p<0.07) and IgG2a (Fig. 4E Group D2, p<0.08) titres relative to the titres generated after a single i.p. immunization. These data indicate that once mucosal tolerance is subverted, it is maintained even after repeated systemic vaccination.

### Newborns Respond to Oral Antigen with Th1-biased Cell-mediated Immunity Indicating Prevention of Mucosal Tolerance

**Figure 5:**
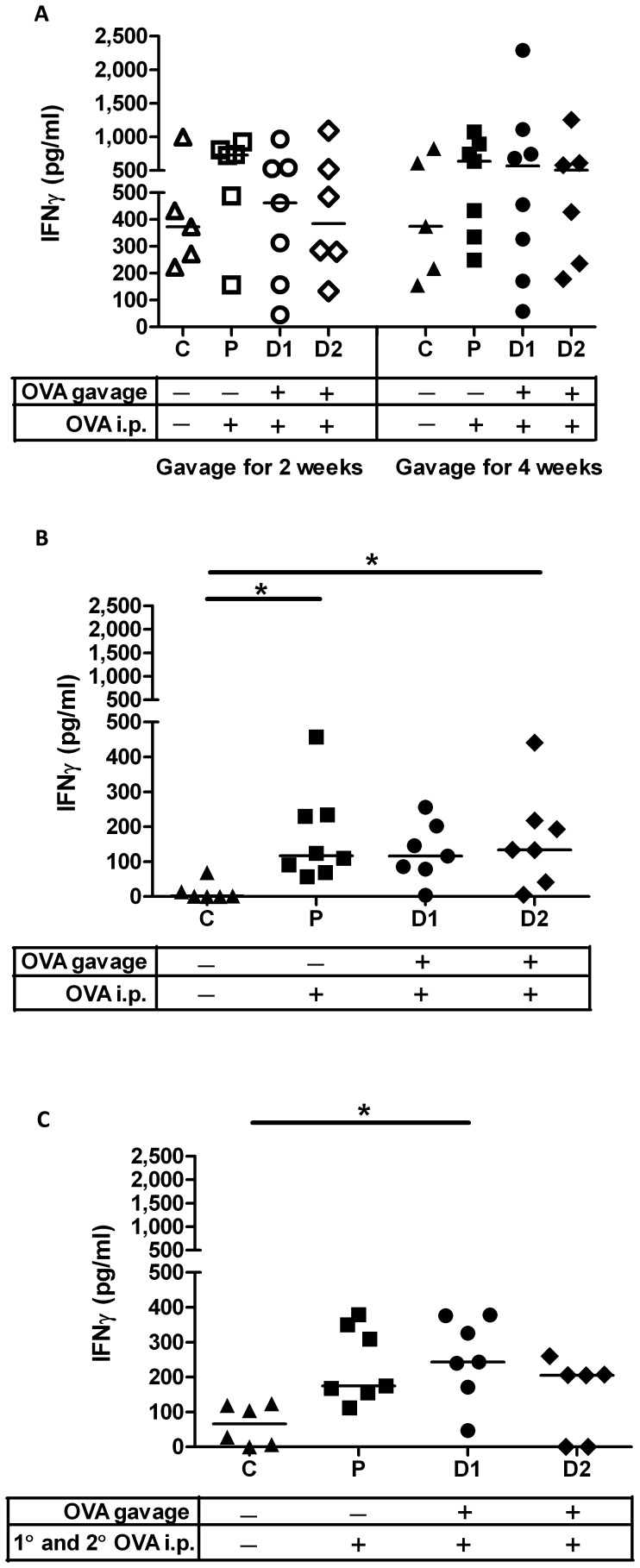
Oral antigen exposure in newborns prevent induction of peripheral tolerance as measured by OVA-specific IFN-γ production by mesenteric lymph nodes. Pups represented in A (newborn pups) and B (older neonatal pups) were treated as detailed in Fig. 1A whereas pups in C were subjected to two i.p. immunizations as detailed in Fig. 1B. IFN-γ produced by *ex vivo* re-stimulated mesenteric lymph node-derived lymphocytes were measured by ELISA 3 weeks after final i.p. immunization. Each data point represents an individual animal and median values are indicated by horizontal lines. *p<0.05.

**Figure 6:**
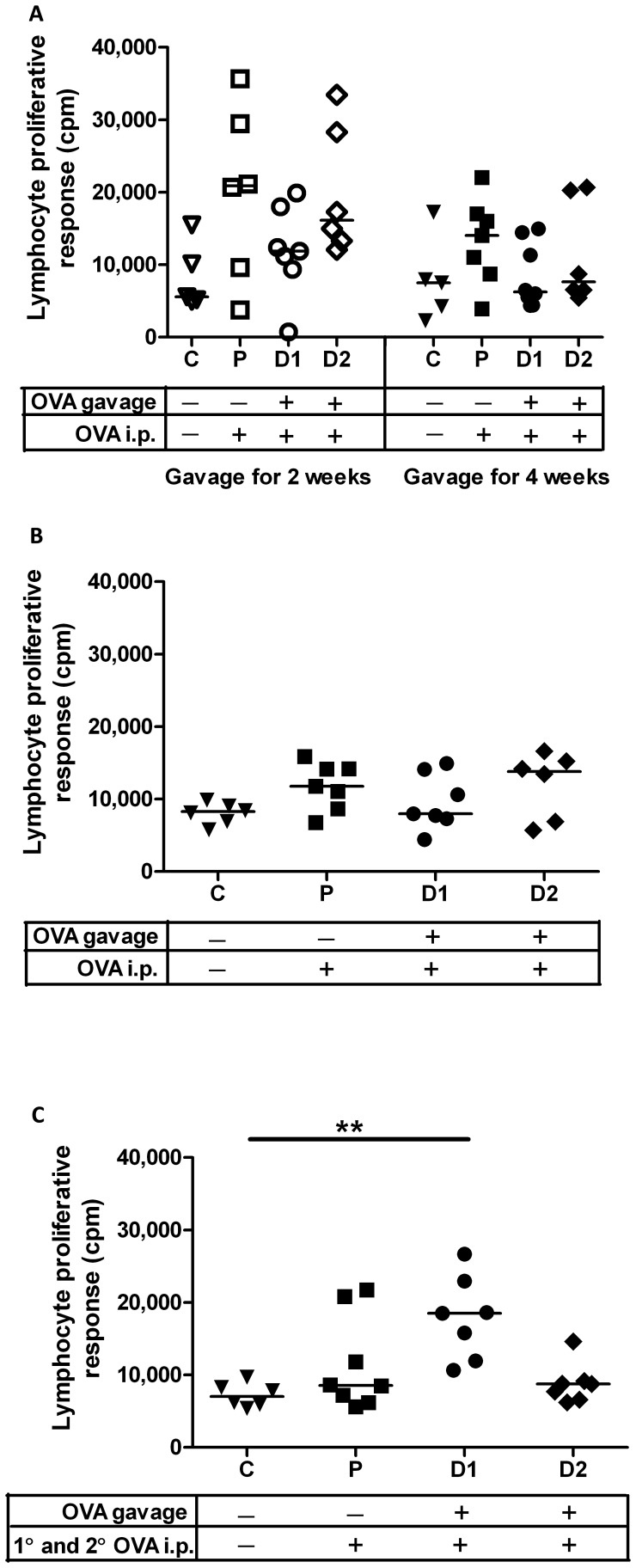
Oral antigen exposure in newborns subjected to repeated i.p. immunizations prevents induction of peripheral tolerance as measured by lymphocyte proliferation. Pups represented in groups A and B were treated as detailed in Fig. 1A whereas pups represented in C were treated as detailed in Fig. 1B. *Ex vivo* re-stimulated mesenteric lymph node-derived lymphocytes were measured by lymphocyte proliferative response. ^3^H-thymidine incorporation was measured and expressed as cpm. Triplicate measures were obtained for each stimulation and the mean value represent the data for each biological replicate. Data presented are individual values and the horizontal line represents the median value for each group. **p<0.01.

To further define the qualitative aspects of oral immunization, draining mesenteric lymph nodes were isolated from all pups from the newborn and older neonatal groups three weeks after the last i.p. immunization. Single-cell suspension of mesenteric lymph node lymphocytes were restimulated with OVA (or saline), *ex vivo* and lymphocyte proliferation and IFN-γ and IL-4 cytokine quantities were assessed. Regardless of dose or duration of gavage, mesenteric lymph node cells obtained from OVA-gavaged newborns as well as parenteral control newborns failed to produce significantly higher IFN-γ titres relative to cells from negative control newborns (Fig. 5A). However, mesenteric lymph node cells obtained from older neonatal group that had been gavaged with 0.1 µg OVA and the parenteral control group showed significantly higher IFNγ relative to the control group (Fig. 5B, D2 p<0.05; P p<0.05). Cells from the newborn oral exposure group subjected to secondary immunization also produced significantly higher IFNγ (Fig. 5C, D1 p<0.05) relative to the age-matched control groups. For all groups of pups, IL-4 was negligible (data not shown). When the lymphocyte proliferative response was assessed, no significant difference was observed between the newborn or older groups relative to their control groups regardless of dose or duration of gavage (Fig. 6A–B). However, lymphocytes from newborn oral exposure group D1 subjected to secondary immunization showed significantly higher proliferation relative to controls (Fig. 6C, D1 p<0.01) relative to the control group. Surprisingly, lymphocytes obtained from parenteral control pups failed to respond with robust lymphocyte proliferation. Together these results suggest that newborn rat pups gavaged with antigen and boosted with primary and secondary systemic exposure prevented induction of oral tolerance and instead promoted antigen-specific cell-mediated, Th1-biased immunity.

Many factors influence whether mucosal tolerance is induced including antigenic dose, timing of exposure, the nature of the antigen and the host’s immunological maturity at time of exposure. Our data corroborates other research which showed that newborn rodents respond to oral antigens with immunity if exposed within the immediate period after birth [9], [10], [37]. Strobel, et al., (1984) demonstrated that a single feeding of a weight-related dose of OVA to mice within the first week of life resulted in priming for both humoral and cell-mediated immune responses, despite the profound tolerance found in adult mice treated in the same way [9]. Miller et al., (1994) determined that rat pups fed antigen via gavage 24 hr and 72 hr after birth were primed for immunity [10]. In a rat model of experimental autoimmune encephalomyelitis, oral administration of myelin basic protein induced priming when given within 3 days after birth but after 4 weeks of age, the same dose invoked tolerance [9], [37]. Hanson, D.G. (1981) determined that 2-day old mice fed a single, body-weight proportioned dose (1 mg/g) of OVA by intragastric intubation showed significant priming of OVA-specific humoral immunity upon subsequent parenteral immunization with OVA in alum [38]. When the antigen was changed from OVA to the readily absorbable human gamma globulins (HGG; 1 mg/g), the mice developed tolerance [38] but they developed an immune response if HGG dose was reduced to 0.1 µg/g. Penttila et al (2012) fed rat pups 1 mg OVA intermittently or daily from day 4 after birth until day 13 [39]. Regardless of the dosing regimen, they determined that dam-reared pups produced low levels of circulating OVA-specific IgE and IgG1 and they suggest that early daily OVA exposure may prevent early allergic sensitization [39]. These data are in excellent agreement with our preliminary work which showed that pups fed 1 mg OVA or higher for up to 2 weeks responded with oral tolerance (referred to in Section 3.1). Together, it is clear that how well an antigen is absorbed and/or the dose may influence whether newborn mice respond with immunity or tolerance.

Tobagus et al, (2004) determined that if Complete Freunds’ Adjuvant was used as the adjuvant during systemic exposure, mice showed induced IL-5 responses but significantly inhibited IgG2α, IL-2, and IFNγ responses [12]. In contrast, when alum was used as adjuvant, the mice showed significant inhibition of IgG1, IgE, IL-2, and IL-5 responses, but increased IFNγ responses. These data suggest that adjuvant type used at the time of systemic immunization may dictate induction of oral tolerance or immune mechanisms.

The maturity of the gut at birth differs across species and therefore may impact how well the animal can respond to antigens. GALT in the intestine of humans [40] and most domestic species displays extensive fetal and neonatal development in the complete absence of commensal microflora [41]. Studies in lambs revealed that *in utero* vaccination resulted in a protective immune response and immune memory confirming that the fetal immune system is mature enough to respond to oral vaccine [42], [43]. Oral inoculation of foals with virulent *Rhodococcus equi* bacteria demonstrated accelerated CTL development and IFN-γ production [44]. Similarly, oral vaccination of newborn piglets with recombinant mutant staphylococcal enterotoxin B resulted in induction of systemic and mucosal immunity [45]. Thus, at the time of birth, the GALT in humans, ruminants and piglets are sufficiently mature to induction protective immunity. In contrast, GALT in the murine small intestine is not active until 5–6 weeks of age [46], [47], MHC class II molecules do not appear in the GALT of the rat until around 4 weeks of age [48], and newborn rodents have an almost complete absence of luminal proteolysis [49] which may impact the generation of altered forms of antigen [38], [50]. Despite the limited maturity of the GALT in newborn rodents, our data and others indicate that antigen exposure within the first 3 days after birth is critical to induce immunity instead of tolerance [9], [10], [38]. Why this brief time period is critical is unclear but it may be due to the lack of established tight-junctions in the gut immediately after birth which results in a semi-permeable gut wall and/or increased pinocytosis by neonatal enterocytes relative to enterocytes in adults [9], [10], [38], [51], [52], [53], [54]. This limited period of increased intestinal permeability allows for the passage of molecules such as maternal antibodies or antigens between or through epithelial cells in an immunologically-intact form [51], [52]. ‘Gut-closure’ occurs after weaning in rodents [20], [21] but in large animal species such as piglets and ruminants, ‘gut closure’ occurs within a few days after birth, significantly reducing maternal antibody uptake across the gut wall after this time [55]. In large animal species such as ruminants, ‘gut closure’ occurs within a few days after birth significantly reducing maternal antibody uptake across the gut wall after this time [55]. Thus, the ‘leaky’ gut wall in the immediate perinatal period may be critical for the induction of specific oral immunity and could possibly facilitate protection against infectious disease. However, other factors besides access to the GALT through a semi-permeable gut-wall may also contribute to the induction of mucosal immunity and/or prevention of oral tolerance. For instance, it may be that tolerogenic DCs and/or Treg cells are not sufficiently mature enough in the neonate to suppress immunity [56]. In our study, if primary or secondary i.p. exposure was delayed until the rat pups reached maturity, we could speculate that tolerogenic DCs or Tregs, which would by that time be mature, may trigger induction of tolerance despite early oral exposure. Alternatively, early oral exposure may set in motion a series of changes in cell recruitment and/or cell signalling events which preferentially block induction of antigen-specific tolerance into adulthood. The critical mechanism(s) for induction of immunity and/or prevention of induction of oral tolerance in extreme early life must be subjected to further examination.

The overwhelming majority of all pathogens invade through the mucosal routes. If a pathogen is encountered but it is of low virulence or it is present in sufficiently low numbers, the pathogen will fail to invade and it will be cleared from the body. As it is being cleared, the mucosal immune system may sample the pathogen and the possibility exists that despite the presence of various pathogen-associated molecular patterns on the pathogen surface, the immune system will induce oral tolerance to one or more pathogenic antigens. Should this occur, subsequent exposure, perhaps by a more virulent strain or larger quantities of the pathogen, will then trigger suppression of immunity which will facilitate, instead of prevent, colonization and infection. However, we propose that if the newborn was proactively orally vaccinated against pathogen-derived antigens in early life, we could subvert induction of mucosal tolerance. Then, upon subsequent mucosal exposure to pathogenic antigens, the immune system would be primed to produce an immune response conferring a significant advantage to the host. Although intriguing, this strategy must be more extensively examined as the nature of the antigen, the dose, the duration of exposure, the timing of initial exposure, and the choice of adjuvants, may all contribute to induction of oral priming versus oral tolerance. Further studies should be performed to establish the characteristics of the DCs which take up the antigen as well as determine whether the antigens are preferentially presented to T cells in Peyer’s Patches or ILF rather than in MLNs. The effects of early life oral priming on induction of auto-immunity must also be carefully evaluated. Whether subversion of oral tolerance and/or induction of mucosal immunity established in newborns protects against disease in later life is currently underway in our laboratory.

### Conclusions

Low dose antigen exposure for a finite period prevents induction of antibody-mediated and cell-mediated tolerance if exposure occurs within the immediate period after birth. Once prevented, induction of mucosal tolerance was maintained into adulthood.
